# Iatrogenic left anterior descending artery stenosis early after aortic valve replacement presenting with T-wave-pseudonormalization - a case report

**DOI:** 10.1186/s13019-015-0239-4

**Published:** 2015-03-21

**Authors:** Stefan Schwarz, Hermann Blessberger, Jürgen Kammler, Christoph Gross, Clemens Steinwender

**Affiliations:** 1 1st Medical Department - Cardiology, Linz General Hospital, Johannes Kepler University School of Medicine, Krankenhausstrasse 9, 4020 Linz, Austria; 2 Department of Cardiothoracic Surgery, Linz General Hospital, Johannes Kepler University School of Medicine, Krankenhausstrasse 9, 4020 Linz, Austria

**Keywords:** Iatrogenic coronary artery stenosis, Aortic valve replacement, Pseudonormalization, Percutaneous coronary intervention

## Abstract

**Background:**

Both iatrogenic coronary artery stenosis early after aortic valve replacement and pseudonormalization of inverted T-waves in acute ischemia are rare but well-recognized findings which coincide in this case.

**Case presentation:**

10 weeks after aortic valve replacement, a 58-year-old male patient was readmitted with recent onset of unstable angina pectoris. Electrocardiography showed inverted T-waves in leads V2-4 with pseudonormalization during episodes of typical chest pain. Coronary angiography revealed subtotal ostial occlusion of the left anterior descending artery which was successfully treated by percutaneous coronary intervention.

**Conclusion:**

Iatrogenic coronary artery stenosis is a potentially life-threatening complication that can occur early after aortic valve replacement in patients with normal preoperative coronary angiography. T-wave-pseudonormalization during episodes of angina pectoris may lead to misinterpretation in patients at high risk.

## Background

Pseudonormalization of abnormal T-waves in spontaneous or provoked ischemia was first described by Noble et al. [1]. The underlying electrophysiological mechanism generally thought to apply is a superposition of acute ischaemic effects on the action potential of subepicardial cells on top of chronic ischaemic effects [2]. Among other probable causes, T-wave inversion in the anterior precordial leads usually reflects severe left anterior descending coronary artery stenosis [3]. Development of angina pectoris within a few months after aortic valve replacement is highly suspicious for iatrogenic proximal coronary artery stenosis [4]. These two rare findings of clinical cardiology coincide in this case.

## Case presentation

We report the case of a 58-year-old male patient undergoing valve replacement surgery for symptomatic aortic valve stenosis. Coronary heart disease had been ruled out preoperatively by coronary angiography. Valve replacement surgery was performed using a St. Jude 25 mm mechanical valve prosthesis (St. Jude Medical Inc., St. Paul, MN, USA). During surgery, cardioplegic solution was administered by selective antegrade ostial cannulation using a 3mm coronary artery perfusion cannula with a 6mm balloon tip (CalMed Laboratories, Costa Mesa, CA, USA). Postoperative recovery and rehabilitation were unremarkable without symptoms of angina pectoris.

**Figure 1:**
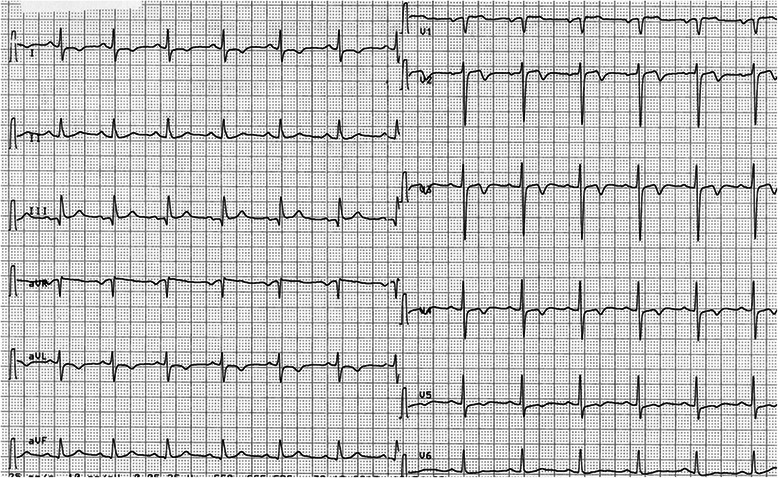
**Standard 12-lead baseline ECG on admission.** Inverted T-waves in leads V2-4.

10 weeks after surgery, the patient was readmitted to our department with recent onset of unstable angina. Oral anticoagulation with phenprocoumon was within therapeutic range with an INR value of 2,7 (target median INR 2,5 in case of low prosthesis thrombogenicity and no patient-related risk factors). Baseline ECG on admission was recorded while the patient was free of symptoms and showed symmetrical negative T-waves in leads V2-4 (Figure 1). Echocardiographic evaluation showed good prosthetic valve function and a normal left ventricular ejection fraction without abnormal regional wall motions.

**Figure 2:**
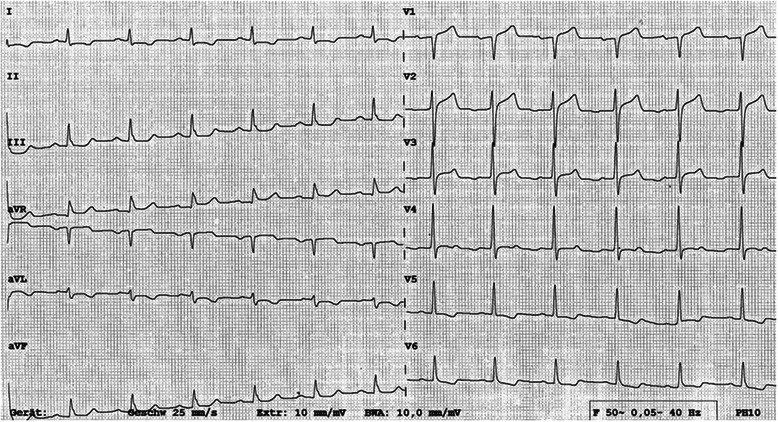
**Pseudonormalization.** Upright T-waves in leads V2-4 during angina pectoris.

Recurrent severe attacks of angina pectoris at rest were successfully treated with morphine and glyceryl trinitrate. Simultaneous ECG recordings showed normalization of the previously inverted T-waves (Figure 2). Despite serial negative measurement of cardiac markers including high sensitive troponin T, loading doses of 250 mg acetylsalicylic acid and 300 mg clopidogrel were administered upon these findings and an early invasive strategy was planned.

**Figure 3:**
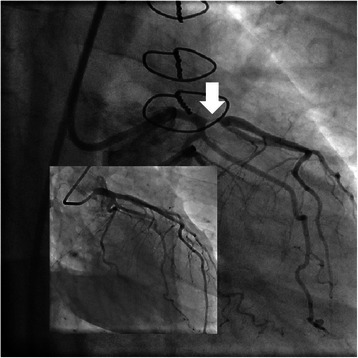
**Subtotal ostial occlusion of the left anterior descending artery (RAO caudal view).** Insert: preoperative coronary angiography.

**Figure 4:**
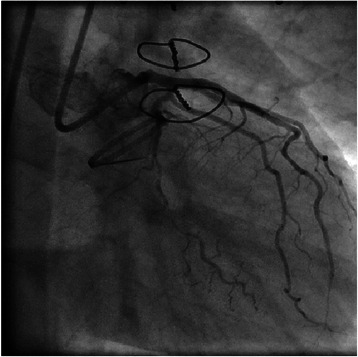
**After percutaneous coronary intervention (RAO caudal view).**

Coronary angiography revealed subtotal ostial occlusion of the left anterior descending artery (Figure 3) and successful percutaneous coronary intervention was performed using a Zotarolimus-eluting stent (Resolute Integrity® 3,0 × 12 mm, Medtronic Inc., Minneapolis, MN, USA) (Figure 4). Antiplatelet therapy will comprise three months of dual antiplatelet therapy (acetylsalicylic acid and clopidogrel) followed by nine months of single antiplatelet therapy (acetylsalicylic acid alone), combined with unaltered continuation of phenprocoumon.

## Discussion

Data concerning the incidence of pseudonormalization in acute coronary syndrome are scarce. In one large perennial single-center survey it was 1% and typically caused by severe coronary artery stenosis lacking coronary collateral development [5]. Pseudonormalization can also occur in the setting of coronary angioplasty, where iatrogenic transient coronary occlusion can serve as a model for understanding acute myocardial ischemia [6]. The electrophysiological basis of T-wave pseudonormalization is the following: chronic ischaemic injury of subepicardial cells causes a longer duration of the action potential than in subendocardial cells and therefore a negative T wave. Pseudonormalization can be seen when acute ischaemic injury in subepicardial cells with previous chronic ischaemic injury will progressively shorten the duration of the action potential compared to subendocardial cells, resulting in an iso-electric or positive T-wave [2]. Knowledge and understanding of this phenomenon should lead to serial ECG recording in patients with typical presentation of unstable angina pectoris.

Iatrogenic coronary artery stenosis (ICAS) occurs after 1 to 5% of aortic valve replacement procedures and the clinical symptoms are usually typical, severe, and appear within the first 6 months following surgery [7,8]. In most cases, the left main stem or the ostium of the right coronary artery are affected, although stenosis of the ostium of the left anterior descending artery has also been reported [9]. Selective antegrade ostial cannulation for administration of cardioplegic solution during surgery is generally thought to cause an initial mechanical vessel wall trauma. Multidetector computed tomography and optical coherence tomography identified fibrous tissue formation and intimal hyperplasia in ICAS, which therefore differs pathophysiologically from conventional atherosclerotic lesions [7,10].

Retrograde administration of cardioplegic solution through the coronary sinus was proposed to prevent the occurrence of ICAS, and, in case of occurence, percutaneous coronary intervention has become an alternative treatment option over coronary bypass surgery, avoiding the risks of early reoperation [8,11].

## Conclusion

Suspicion of iatrogenic coronary artery stenosis early after aortic valve replacement should lead to prompt invasive management in patients presenting with unstable angina pectoris and dynamic ECG-changes, whereas cardiac computed tomography could be the next diagnostic step in the absence of dynamic ECG-changes, especially if cardiac markers are negative. Pseudonormalization can mask otherwise obvious pathological T-waves when ECG is recorded during acute chest pain and is a potential diagnostic pitfall.

## Consent

Written informed consent was obtained from the patient for publication of this case report and any accompanying images. A copy of the written consent is available for review by the Editor-in-Chief of this journal.

## Abbreviations

AVR: Aortic valve replacement
ICAS: Iatrogenic coronary artery stenosis
